# The effect of Nickel hypersensitivity on the outcome of total knee arthroplasty and the value of skin patch testing: a systematic review

**DOI:** 10.1186/s42836-022-00144-5

**Published:** 2022-09-02

**Authors:** C. J. H. Peacock, H. Fu, V. Asopa, N. D. Clement, D. Kader, D. H. Sochart

**Affiliations:** 1 South West London Elective Orthopedic Centre, Epsom, UK; 2 Edinburgh Orthopedics, Royal Infirmary of Edinburgh, Edinburgh, UK; 3 The School of Health and Society, The University of Salford, Salford, UK

**Keywords:** Nickel hypersensitivity, Total knee arthroplasty, Total knee replacement, TKA, TKR, Patch testing

## Abstract

**Background:**

To assess the Nickel sensitizing potential of total knee arthroplasty (TKA), explore the relationship between hypersensitivity and clinical outcomes, and evaluate the utility of skin patch testing pre- and/or postoperatively.

**Materials and methods:**

A literature search was performed through EMBASE, Medline and PubMed databases. Articles were screened independently by two investigators. The level of evidence of studies was assessed using the Oxford Centre for Evidence-Based Medicine Criteria and the quality evaluated using the Methodological Index for Non-randomized Studies and Cochrane risk-of-bias tools.

**Results:**

Twenty studies met the eligibility criteria, reporting on 1354 knee arthroplasties. Studies included patients undergoing primary or revision TKA, pre- and/or postoperatively, and used patch testing to identify Nickel hypersensitivity. Prevalence of Nickel hypersensitivity ranged from 0% to 87.5%. One study compared the prevalence of Nickel hypersensitivity in the same patient group before and after surgery and noted newly positive patch test reactions in three patients (4.2%). Three studies reported lower prevalence of Nickel hypersensitivity in postoperative patients compared to preoperative ones. Seven studies suggested that hypersensitivity might cause adverse clinical outcomes, but six did not support any relationship. Seven studies recommended preoperative patch testing in patients with history of metal allergy, and nine concluded that testing may be valuable postoperatively.

**Conclusions:**

Patients undergoing TKA with no prior history of metal hypersensitivity do not seem to be at an increased risk of developing Nickel hypersensitivity, and there is conflicting evidence that patients with pre-existing hypersensitivity are more likely to experience adverse outcomes. Patch testing remains the most commonly used method for diagnosing hypersensitivity, and evidence suggests preoperative testing in patients with history of metal allergy to aid prosthesis selection, and postoperatively in patients with suspected hypersensitivity once common causes of implant failure have been excluded, since revision with hypoallergenic implants may alleviate symptoms.

**Supplementary Information:**

The online version contains supplementary material available at 10.1186/s42836-022-00144-5.

## Background

The reported prevalence of metal hypersensitivity in the general population ranges from 10 to 15% [1]. Nickel hypersensitivity is the most common, followed by Chromium and Cobalt, with approximately 14% of the general population having cutaneous sensitivity to Nickel [1]. The prevalence is reported to be four times more prevalent in females [2] and in certain occupations such as hairdressing, catering and bar work [3, 4]. Total knee arthroplasty (TKA) implants are typically composed of Nickel, Cobalt, Chromium, Molybdenum, Zirconium and Titanium alloys [5], and it has been suggested that patients could develop hypersensitivity reactions to these metals and associated complications postoperatively [6]. In total hip arthroplasty (THA), the prevalence of metal hypersensitivity has been reported to be approximately 25% in patients with well-functioning implants and up to 60% in those with failed or poorly functioning implants [5]. Similarly, the reported prevalence of metal hypersensitivity in TKA patients with stable implants is 44%, and 57% in those with loosened implants [7]. However, it remains uncertain whether the relationship between sensitization and implant failure is cause or effect.

Patients with metal hypersensitivity can present in a similar way to joint infection [1, 8]. Symptoms may include persistent pain, swelling and stiffness, with the onset of symptoms occurring between 2 months and 2 years following primary TKA [1, 9]. The patient may develop localized dermatitis, effusions, and reduced range of motion [8, 10]. More generalized eczematous reactions, though less common, can occur [11]. Radiography is typically unremarkable but might demonstrate periprosthetic osteolysis or implant loosening [12].

Metal hypersensitivity is a diagnosis of exclusion once more common causes of implant failure, such as infection and aseptic loosening, have been ruled out [10, 13]. Currently, there is no established or reliable test for detecting metal hypersensitivity, although skin patch testing (PT) is often employed due to ease of application, widespread availability, breadth of evaluation, and rapidity of results [9, 10, 14]. However, there is a lack of consensus over the clinical utility of patch testing patients with TKA [6, 10].

Since metal hypersensitivity occurs most frequently from exposure to Nickel, this systematic review was performed to collate and analyze the current literature on Nickel hypersensitivity in patients undergoing TKA. Previous review articles [1, 5, 6, 9, 10, 13–19] have been published providing an overview of metal hypersensitivity in total joint arthroplasty, but the current review focuses specifically on Nickel hypersensitivity in TKA patients as well as the usefulness of patch testing. The aims of the study were to evaluate: (1) the Nickel sensitizing potential of TKA, (2) the relationship between Nickel hypersensitivity and clinical outcomes and (3) the utility of skin patch testing in TKA patients pre- and/or postoperatively.

## Materials and methods

This review was conducted in accordance with the 2020 Preferred Reporting Items for Systematic Review and Meta-Analysis (PRISMA) guidelines [20].

### Eligibility criteria

Clinical studies determining the prevalence of Nickel hypersensitivity by patch testing patients with total knee arthroplasty, pre- and/or postoperatively, were included. Studies which also involved patients undergoing orthopedic interventions other than TKA were not excluded, provided that an appropriate number of TKA patients were included. Full-text articles had to be available and published in English or with translation freely available. Case reports, review articles, conference abstracts and surveys were excluded.

### Search strategy

A comprehensive electronic search strategy utilizing a combination of Medical Subject Heading (MeSH)-terms and keywords was developed by one author (CP) and refined with the help of the Department Librarian (PA). The EMBASE and Medline databases were searched using the Healthcare Database Advance Search (HDAS) platform and extended to the native PubMed database, identifying literature from inception until September 2021. The line-by-line strategy run in HDAS and PubMed is outlined in Supplementary Material 1 and Supplementary Material 2, respectively. The only limitation to the search strategy was the ‘search field’, restricted to title and abstract, ensuring the literature search was sensitive and yielded all articles meeting the eligibility criteria.

To supplement the electronic search, a detailed review of the reference lists of the final studies included in the systematic review and in review articles on the same or similar topic was performed. Finally, a search of the grey literature on OpenGrey was performed to identify any published or ongoing research.

### Screening

Potentially eligible studies were identified by screening the titles and abstracts of all articles retrieved from the search. The eligibility of each full-text article was then assessed for inclusion. Each stage was performed independently by two investigators (CP, HF), and any inconsistencies were discussed until consensus obtained. Disagreements at either stage were resolved by the senior author (DHS).

### Data extraction/Analysis

The following data were extracted from the included studies:

- Study characteristics (*e.g*. author, year, country, *etc*.)
- Patient characteristics (*e.g*. sample size, average age, percentage of females *etc*.)
- Type of TKA implant (*i.e*. metallic composition)
- Details of patch testing (*i.e*. composition, timing)
- Prevalence of Nickel hypersensitivity (*i.e*. number of patients, percentage of population)
- Relevant clinical results (*e*.*g*. complications, implant status, further management *etc*.)
- Main conclusions and recommendations

A data collection table in Microsoft Excel was designed by one author (CP) to display the information extracted from each eligible study.

Owing to heterogeneity in study design, participants, interventions and outcome measures, a quantitative meta-analysis was not appropriate.

### Methodological quality assessment

Levels of evidence (LE) were assessed using the Oxford Centre for Evidence-Based Medicine (OCEBM) framework [21].

The quality of observational studies was independently assessed by two authors (CP, HF) using the Methodological Index for Non-randomized Studies (MINORS) tool [22]. The ideal global score was 16 for non-comparative studies and 24 for comparative studies.

Any randomized controlled trials were scored using the revised Cochrane risk-of-bias tool for randomized trials (RoB 2) [23].

The assessments provided an overall impression of each study but were not used to weight any studies in the analysis.

## Results

### Search results

**Fig. 1:**
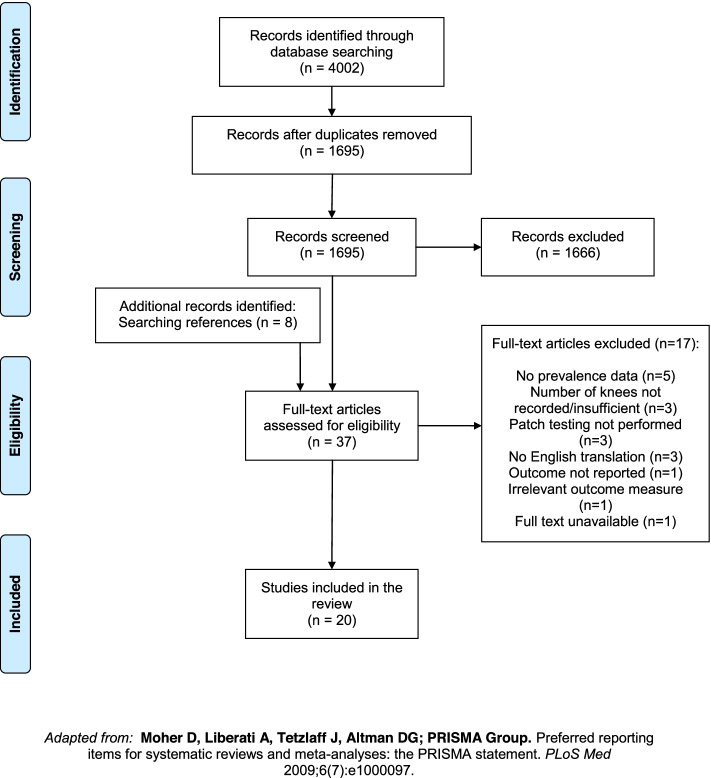
PRISMA flow diagram for search results

The initial search performed through the EMBASE, Medline and PubMed databases identified 4002 records, of which 1695 records remained after deduplication. Of these, 1666 were excluded after screening titles and abstracts. Eight additional studies were identified by searching the reference lists of articles on the same topic, and no studies were identified by performing a search of the grey literature. As a result, 37 full-text articles were assessed for eligibility for inclusion, of which 17 were excluded (Fig. 1). Twenty studies were deemed eligible for inclusion in the review.

### Quality assessment

Nineteen observational studies were included, four having a cohort study design with LE of III [24–27], and 15 being either case-control or case-series, with LE of IV [7, 28–41]. The average MINORS score was 9.75 and 14.29 for non-comparative and comparative observational studies respectively. There was one randomized-controlled trial (RCT) with LE of II [42] (Table 1).

**Table 1:** Study design, level of evidence (LE) and Quality Assessment Score (MINORS for observational studies, RoB 2 for randomized controlled trials) for individual studies

**Author**	**Study design**	**LE**	**Quality Assessment**
Atanaskova Mesinkovska *et al*. [24]	Cohort	III	MINORS 14/24
Carlsson and Möller [25]	Cohort	III	MINORS 10/16
Carossino *et al*. [28]	Case–control	IV	MINORS 15/24
Desai *et al*. [29]	Case-series	IV	MINORS 12/16
Frigerio *et al*. [30]	Case-series	IV	MINORS 10/16
Granchi *et al*. [7]	Case–control	IV	MINORS 22/24
Guenther *et al*. [31]	Case-series	IV	MINORS 10/16
Innocenti *et al*. [32]	Case-series	IV	MINORS 10/16
Kitagawa *et al*. [26]	Cohort	III	MINORS 18/24
Kręcisz *et al*. [27]	Cohort	III	MINORS 11/16
Lützner *et al*. [42]	RCT	II	RoB 2—high risk of bias
Sasseville *et al*. [33]	Case-series	IV	MINORS 11/16
Tam *et al*. [34]	Case-series	IV	MINORS 11/16
Thomas *et al*. [35]	Case-control	IV	MINORS 11/24
Thomas *et al*. [36]	Case-series	IV	MINORS 12/16
Thomas *et al*. [37]	Case-control	IV	MINORS 9/24
Treudler and Simon [38]	Case-series	IV	MINORS 8/16
Verma *et al*. [39]	Case-series	IV	MINORS 3/16
Webley *et al*. [40]	Case-control	IV	MINORS 11/24
Zeng *et al*. [41]	Case-series	IV	MINORS 9/16

*Notes. RCT* Randomized controlled trial, *RoB* Risk of bias

### Study characteristics

Details of the characteristics of each individual study are shown in Table 2.

**Table 2:** Study characteristics with year, country, number of patients, number of total knee arthroplasties (TKAs), mean age (range or SD), and proportion of females (%)

**Author**	**Year**	**Country**	**No. patients**	**No. TKAs**	**Mean age (range or SD)**	**Proportion of females**
Atanaskova Mesinkovska *et al*. [24]	2012	USA	72	31	57 (14–81)	64%
Carlsson and Möller [25]	1989	Sweden	18	3	NR	NR
Carossino *et al*. [28]	2016	Italy	39	30	NR	NR
Desai *et al*. [29]	2019	India	233	233	60 (30–78)	64%
Frigerio *et al*. [30]	2011	Italy	100	52	68 (51–84)	73%
Granchi *et al*. [7]	2008	Italy	94	74	68 (± 8.0)	71%
Guenther *et al*. [31]	2016	Germany	17	14	58 (± 9.8)	100%
Innocenti *et al*. [32]	2014	Italy	24	25	73 (54–86)	71%
Kitagawa *et al*. [26]	2013	Japan	48	48	NR (64–89)	88%
Kręcisz *et al*. [27]	2012	Poland	60	21	62 (NR)	72%
Lützner *et al*. [42]	2013	Germany	120	120	67 (± 8.7)	56%
Sasseville *et al*. [33]	2021	USA	39	45	63 (± 9.7)	41%
Tam *et al*. [34]	2020	USA	127	39	55 (11–90)	74%
Thomas *et al*. [35]	2015	Germany	45	37	65 (37–75)	58%
Thomas *et al*. [36]	2015	Germany	250	189	65 (37–84)	66%
Thomas *et al*. [37]	2013	Germany	368	234	65 (18–96)	67%
Treudler and Simon [38]	2007	Germany	13	13	63 (42–94)	69%
Verma *et al*. [39]	2006	India	15	15	65 (65–80)	87%
Webley *et al*. [40]	1978	UK	83	83	65 (44–76)	77%
Zeng *et al*. [41]	2014	China	96	48	53 (± 15.4)	59%

*Notes. SD* Standard deviation

The 20 studies included a total of 1354 TKAs, with an average of 68 knees per study. Amongst the studies which provided the mean age of all the participants involved (three studies with missing data [25, 26, 28]), the average age was 63.1 years (range, 11–96). The average proportion of females was 70% (two studies with missing data [25, 28]).

### Patient characteristics

All patients underwent primary or revision TKA, with the other study participants comprising either control groups (*e*.*g*. no implant) or undergoing a different surgical procedure, such as THA. Fifteen studies [7, 25, 26, 30–33, 35–42] recorded the type of TKA implant used, with 13 noting the metallic composition of the prosthesis [7, 25, 26, 30–33, 35–39, 42]. The remaining five studies did not clearly document the type of implant used [24, 27–29, 34]. The characteristics of each individual patient group, including sample size, mean age, percentage of females, and implant type, are outlined in Table 3.

**Table 3:** Characteristics of individual patient groups in each study with sample size (*n*), mean age (range or SD), number of females (proportion) and type of TKA implant received

**Study**	**Patient group**	***n***	**Mean age (range or SD)**	**No. females (%)**	**Type of TKA implant**
Atanaskova Mesinkovska *et al*. [24]	Preop patients with potential metal hypersensitivity before implantation of an orthopaedic metal device	31	56.1 (± 15.4)	23 (74%)	-
	Postop patients with potential metal hypersensitivity after implantation of an orthopedic metal device	41	56.8 (± 16.5)	23 (56%)	NR
Carlsson and Möller [25]	Patients with contact allergy to Chromium, Cobalt and/or Nickel (verified by patch test preop) followed up after implantation of various metallic orthopedic devices (3 TKA, 15 other orthopedic implants) containing metal to which they were allergic	18	NR	NR	2 patients – CrCoNi 1 patient – CoCr
Carossino *et al*. [28]	Control group – no implant, no skin/immunological/metabolic or chronic disease	9	NR	NR	-
	Patients awaiting TKA with documented clinical history of metal allergy and hypersensitivity reactions	8	NR	NR	-
	Postop TKA patients with pain with referred metal allergy	11	NR	NR	NR
	Postop TKA patients with pain with no referred metal allergy	11	NR	NR	NR
Desai *et al*. [29]	Postop TKA patients	233	59.6 (30–78)	149 (64%)	NR
Frigerio *et al*. [30]	TKA patients assessed pre- and postoperatively	52	NR	NR	33 patients – Femur: CoCrMo; Tibia: TiAlV 10 patients—CoCrMo 9 patients—TiAlV
	THA patients assessed pre- and postoperatively	48	NR	NR	-
Granchi *et al*. [7]	Control group – no implant, candidates for TKA	20	65.2 (42–84)	14 (71%)	-
	Postop TKA patients with stable implant	27	66.1 (42–84)	22 (82%)	23 patients – Femur: CoCrMo; Tibia: TiAlV 3 patients—CoCrMo 1 patient—TiAlV
	Postop TKA patients with loosened implant	47	70.4 (57–79)	31 (66%)	27 patients – Femur: CoCrMo; TiAlV 16 patients—CoCrMo 2 patients—TiAlV 2 patients—unknown
Guenther *et al*. [31]	Historic database patients with preoperative known sensitisation to Chromium, Cobalt, Nickel, or cement component who underwent revision knee (*n* = 14) and hip (*n* = 3) arthroplasty due to a potential allergic reaction	17	58.2 (± 9.8)	17 (100%)	7 patients—unknown bicondylar surface replacement 3 patients – CoCrMo, UHMWPE 2 patients – CoCrMo, TiAlVa, UHMWPE 1 patient – OxZr 1 patient—CoCr, UHMWPE
Innocenti *et al*. [32]	Preop TKA patients with referred or suspected metal allergy receiving a non-allergenic implant	24	72.9 (54–86)	17 (71%)	Femur: OxZr; Tibia: All-polyethylene
Kitagawa *et al*. [26]	Patients before and after TKA with CoCr or OxZr implants	48	NR (64–89)	42 (88%)	25 patients—Femur: CoCr; Tibia: TiAlVa; Polyethylene insert 22 patients—Femur: OxZr; Tibia: TiAlVa; Polyethylene insert 1 patient—ceramic implant
Kręcisz *et al*. [27]	Preop TKA patients	21	NR	16 (76%)	-
	Preop THA patients	39	NR	27 (69%)	-
	Postop TKA or THA patients	48	NR	36 (75%)	NR
Lützner *et al*. [42]	Patients awaiting TKA randomly assigned to receive coated hypoallergenic implant	61	65.6 (± 9.1)	33 (54%)	CoCrMo with multilayer coating system (Cr, CrN-CrCN, ZrN)
	Patients awaiting TKA randomly assigned to receive standard implant	59	68.1 (± 8.2)	34 (59%)	CoCrMo
Sasseville *et al*. [33]	Postop TKA patients with complications	39	63.3 (± 9.7)	16 (41%)	13 patients—Stainless steel 13 patients—Missing data 5 patients—Ti 2 patients—CoCr, Ti 1 patient – OxZr 1 patient – OxZr, Ti 1 patient—CoCr 1 patient—Ceramic 1 patient—Stainless steel × 2 1 patient—Stainless steel and OxZr
Tam *et al*. [34]	Pre-op patients referred for evaluation of MHS before implantation of orthopaedic (*n* = 21), cardiovascular (*n* = 7), dental (*n* = 8) and other (*n* = 4) devices (12 TKA patients)	40	48.7 (11–90)	32 (80%)	NR
	Postop patients referred for evaluation of MHS after implantation of orthopaedic (*n* = 49), cardiovascular (*n* = 4), dental (*n* = 28) and other (*n* = 6) devices (27 TKA patients)	87	58.3 (14–85)	62 (71%)	NR
Thomas *et al*. [35]	TKA patients with yet unexplained complications (loosening, recurrent effusions, and pain)	25	63.0 (37–75)	9 (36%)	CoCrMo
	"OA-control group" – OA patients awaiting TKA	12	69.2 (52–89)	11 (92%)	-
	"PT-control group" – patients without implant but having undergone patch testing for suspected skin allergy	8	64.3 (53–75)	6 (75%)	-
Thomas *et al*. [36]	TKA (*n* = 189) and THA (*n* = 61) patients suspected of having allergic reactions with complaints of pain (90.5%), reduced ROM (74%), swelling (67.5%), effusions (29%), loosening (16.5%) and eczema (5.5%)	250	64.8 (37–84)	164 (66%)	CoCrMo
Thomas *et al*. [37]	Patients with eczema without metal implant, no CMI	30	52.4 (18–75)	8 (27%)	-
	Patients with eczema without metal implant, with CMI	38	61.6 (44–75)	34 (89%)	-
	Postop TKA (*n* = 43) and THA (*n* = 53) patients without symptoms/complications	100	72.4 (29–96)	75 (75%)	CoCrMo
	Postop TKA (*n* = 187) and THA (*n* = 13) patients with symptoms/complications	200	64.4 (37–84)	130 (65%)	CoCrMo
Treudler and Simon [38]	Postop TKA patients with suspicion of contact allergy to implant material	13	62.8 (42–94)	9 (69%)	11 patients—CoCrMo 2 patients—Ti
Verma *et al*. [39]	Postop TKA patients with eczema surrounding the knee	15	65 (65–80)	13 (87%)	Femur: CoCrMo Tibia: TiAlV
Webley *et al*. [40]	Control group – patients with rheumatoid arthritis or osteoarthritis without prostheses	33	64 (47–76)	26 (79%)	-
	Postop patients with hinge arthroplasty of the knee investigated for possible metal sensitivity	50	66 (44–76)	38 (76%)	Walldius or Guepar type hinge arthroplasty
Zeng *et al*. [41]	Patients undergoing TKA and monitored for post-operative pain	29	65.1 (± 9.2)	25 (86%)	25 patients – Gemini MKII PS 4 patients – NR
	Patients undergoing THA and monitored for postoperative pain	67	48.3 (± 14.9)	32 (48%)	-

*Notes. Al* Aluminium, *CMI* Cutaneous metal intolerance, *CN* Carbonitride, *Co* Cobalt, *COC* Ceramic-on-ceramic, *COP* Ceramic-on-plastic, *Cr* Chromium, *LTT* Lymphocyte transformation testing, *Mo* Molybdenum, *MOP* Metal-on-plastic, *N* Nitride, *NR* Not recorded, *Ox* Oxidized, *PS* Posterior stabilized, *SD* Standard deviation, *THA* Total hip arthroplasty, *Ti* Titanium, *TJA* Total joint arthroplasty, *TKA* Total knee arthroplasty, *UHMWPE* Ultra High Molecular Weight Polyethylene, *V* Vanadium, *Zr* Zirconium

### Patch testing

All 20 studies used patch testing to identify metal hypersensitivity. Details, including the composition and timing of testing in each study, are outlined in Table 4. The substances applied in the patch test, including the precise concentration of Nickel antigen, were documented in 18 studies (Table 4). Thirteen studies used Nickel Sulphate 5% [7, 25–30, 32, 36, 38–40, 42], one used Nickel Sulphate 2.5% [33], and two used both 2.5% and 5% [24, 34]. Three studies did not record the concentration of Nickel Sulphate used [35, 37, 41], while one did not document any of the substances used [31].

**Table 4:** Patch test composition and timing of testing for each study

**Study**	**Patch test composition**	**Timing of testing**
Atanaskova Mesinkovska *et al*. [24]	Nickel Sulphate 5%, Nickel Sulphate 2.5%, 4,4-Diaminodiphenylmethane 0.5%, Aluminum Chloride, Ammonium Tetrachloroplatinate 0.25%, Ammonium Vanadate 1%, Ammonium Heptamolybdate 1%, Bacitracin 20%, Benzoyl Peroxide 1%, Chlorhexidine Digluconate 0.5%, Cobalt (II) Chloride Hexahydrate 1%, Colophony 20%, Copper Sulphate 2%, Ferrous Chloride 2%, Ferrous Sulphate 5%, Formaldehyde 1%, Gentamicin Sulfate 20%, Gold Sodium Thiosulphate 0.5%, Hydroquinone 1%, Indium (III) Chloride 1%, Iridium 1%, Iridium (III) Chloride 1%, Manganese Chloride 2%, Methyl Methacrylate 2%, N,N-Dimethyl-4-toluidine 2%, Neomycin Sulphate 20%, Palladium Chloride 2%, Polyethylene disc, Potassium Dichromate 0.25%, Tantal 1%, Thimerosal 0.1%, Tin (II) Chloride 0.5%, Titanium Dioxide 10%, Titanium disc, Titanium (IV) Oxide 0.1%, Titanium powder 1%, Tobramycin 20%, Vanadium 5%, Vancomycin 0.005%, Zirconium (IV) Oxide 0.1%	Preoperatively Postoperatively (median follow-up 21 months, range 1–232)
Carlsson and Möller [25]	Nickel Sulphate 5%, Cobalt Chloride 1%, Potassium Dichromate 0.5%	Postoperatively (mean follow-up 6.3 years, range 1–16)
Carossino *et al*. [28]	Nickel Sulphate 5%, Chromium III 2%, Cobalt Chloride 1%, Potassium Dichromate 0.5%	Preoperatively Postoperatively (after at least 6 months)
Desai *et al*. [29]	Nickel Sulphate 5%, Cobalt Sulphate 5%, Potassium Bichromate 0.1%	Postoperatively (after at least 3 months)
Frigerio *et al*. [30]	Nickel Sulphate 5%, Cobalt Chloride 1%, Copper Sulphate 2%, Molybdenum 5%, Palladium 2%, Potassium Dichromate 0.5%, Silver Nitrate 1%, Tin 50%, Titanium 10%, Vanadium 5%	Preoperatively Postoperatively (after 1 year)
Granchi *et al*. [7]	Nickel Sulphate 5%, Aluminium Chloride 1%, Chromium Trichloride 2%, Cobalt Chloride 1%, Ferric Chloride 2%, Manganese Chloride 2%, Molybdenum Chloride 2%, Niobium Chloride 1%, Potassium Dichromate 0.5%, Titanium Dioxide 2%, Vanadium Trichloride 2%	Preoperatively Postoperatively (Stable TKA: median follow-up 18 months, range 9.6–120; loosened TKA: median follow-up 24 months, range 4.8–132)
Guenther *et al*. [31]	NR	Postoperatively (mean follow-up 2 years)
Innocenti *et al*. [32]	Nickel Sulphate 5%, Chromium III, Cobalt Chloride 1%, Potassium Dichromate 0.5%, Vaseline	Preoperatively
Kitagawa *et al*. [26]	Nickel Sulphate 5%, Aluminium Chloride 2%, Chromium Trichloride 2%, Cobalt Chloride 2%, Molybdenum Chloride 5%, Titanium Dioxide 10%, Vanadium Trichoride 5%	Preoperatively Postoperatively (after 6 months)
Kręcisz *et al*. [27]	Nickel Sulphate 5%, Aluminium 100%, Ammonium Molybdate Tetrahydrate 1%, Cobalt Chloride 1%, Copper Sulphate 2%, Molybdenum 5%, Palladium Chloride 2%, Potassium Dichromate 0.5%, Vanadium 5%, Vanadium Chloride 1%, Titanium Oxide 10%	Preoperatively Postoperatively (after 24 months)
Lützner *et al*. [42]	Nickel Sulphate 5%, Cobalt Chloride 1%, Molybdenum(V) Chloride 0.5%, Potassium Dichromate 0.5%	Preoperatively Postoperatively (after 1 year)
Sasseville *et al*. [33]	Nickel Sulphate 2.5%, 2-Hydroxyethyl Methacrylate, Cobalt Chloride Hexahydrate 1%, Ethyl Acrylate 0.1%, Methyl Methacrylate 2%, Neomycin 20%, Potassium Dichromate 0.25%	Postoperatively (mean follow-up 29.1 months, SD 20.1)
Tam *et al*. [34]	North American baseline series of 50 allergens and custom series (*e.g.* metal series, dental series, bone cement series) based on clinical history Nickel Sulphate 5%, Nickel Sulphate 2.5%, Cobalt Chloride 1%, Gold Sodium Thiosulfate 0.5/2%, Iridium Chloride 10%, Manganese Chloride 2%, Mercuric Chloride 0.1%, Mercury 0.5%, Mercury Ammonium Chloride 1%, Palladium Chloride 2%, Potassium Dichromate 0.25%, Potassium Dicyanoaurate 0.1%, Stannous Chloride 1%, Vanadium 5%, Zinc Chloride 2%	Preoperatively Postoperatively (time frame NR)
Thomas *et al*. [35]	Standard series with 30 allergens (includes Nickel, Chromium, Cobalt), metal allergen series, and bone cement series	Postoperatively (time frame NR)
Thomas *et al*. [36]	Baseline series with 29 allergens (includes Nickel Sulphate 5%), routine supplemental series, and bone cement component series	Postoperatively (time frame NR)
Thomas *et al*. [37]	Standard series (includes Nickel, Chromium, Cobalt), additional series adapted to exposure history, and bone cement series	Postoperatively (time frame NR)
Treudler and Simon [38]	Nickel Sulphate 5%, benzoyl peroxide 1%, Cobalt Chloride 1%, Copper Sulphate 1%, Gentamicin 20%, Hydroquinone 1%, Hydroxyethyl Methacrylate 1%, Manganese Chloride 0.5%, Molybdenum Chloride 2%, Potassium Dichromate 0.5%, Titanium Oxide 0.1%, Vanadium Pentoxide 10%	Postoperatively (average follow-up NR, range 6–36 months)
Verma *et al*. [39]	Nickel Sulphate 5%, Cobalt Chloride 1%, Potassium Dichromate 0.5%	Postoperatively (time frame NR)
Webley *et al*. [40]	Nickel Sulphate 5%, Acrylic Polymer 1%, Acrylic 10%, Cement 1% and 10%, Cobalt Chloride 2%, Iron 2%, Manganese 2%, Molybdenum 1%, Potassium Dichromate 0.5%, Silicon 2%	Postoperatively (mean follow-up 2.7 years, range 1–5 years)
Zeng *et al*. [41]	Nickel, Cobalt, Chromium, Aluminium, Copper, Iron, Manganese, Molybdenum, Tin, Titanium, Vanadium, Zirconium	Preoperatively

*Notes. NR* Not recorded, *SD* Standard deviation, *TKA* Total knee arthroplasty

In four studies [26, 27, 30, 42], the same group of participants underwent patch testing before and after surgery. Four studies [7, 24, 28, 34] performed patch testing on one cohort of patients preoperatively and on a different cohort postoperatively. In ten studies [25, 29, 31, 33, 35–40], patients were patch tested only postoperatively, whilst in two [32, 41] patch testing was performed only preoperatively. The reported time until patch testing was performed postoperatively ranged from 3 months [29] to 16 years [25], but five studies [34–37, 39] did not record when the postoperative patch testing was performed.

### Prevalence of Nickel hypersensitivity

The prevalence of Nickel hypersensitivity in the individual populations of each study together with relevant clinical outcomes, such as complications, implant status, and further management, is summarized in Table 5. The prevalence of Nickel hypersensitivity across the studied populations ranged from 0% [7, 26, 37] to 87.5% [28]. Four studies [26, 27, 30, 42] analyzed the prevalence in the same patient group before and after surgery. One study [30] noted that three patients (4.2%) who tested negative initially developed a newly positive reaction to Nickel after their operation. One study [26] noted that no patients had developed a newly positive reaction compared to their preoperative baseline. One study [27] did not record a significant increase in prevalence following surgery and another [42] noted that two patients had developed ‘doubtful’ patch test reactions.

**Table 5:** Prevalence of Nickel hypersensitivity across the studies together with relevant clinical outcomes for each population (*e.g.* complications, status of implant, further management *etc*.)

**Study**	**Timing of testing**	**Population**		**Sample size (*n*)**	**Nickel hypersensitivity**		**Clinical results (***e.g.* complications, status of implant, further management *etc*.)
					**No. patients**	**Proportion**	
Atanaskova Mesinkovska *et al*. [24]	Preoperatively	Before implantation of an orthopedic metal device		31	16^a^	52.0%	Patients with metal hypersensitivity received a hypoallergenic implant and developed no complications attributable to hypersensitivity at time of follow-up
	Postoperatively (median follow-up 21 months, range 1–232)	After implantation of an orthopedic metal device		41	10^a^	24.0%	6 out of 10 patients with positive patch test to a metal in their implant had the prosthesis removed leading to resolution of symptoms. The other 4 patients did not undergo revision surgery and continued to experience symptoms.
Carlsson and Möller [25]	Postoperatively (mean follow-up 6.3 years, range 1 to 16 years)	Patients with contact allergy to Chromium, Cobalt and/or Nickel (verified by patch test preop) followed up after implantation of various metallic orthopedic devices (3 TKA, 15 other orthopedic implants) containing metal to which they were allergic ^c^		18	15^a^/^b^	83.3%	No patients developed dermatologic or orthopedic complications attributable to contact allergy
Carossino *et al*. [28]	-	Control group—no implant, no skin/immunological/metabolic or chronic disease		9	NR	NR	
	Preoperatively	Patients awaiting TKA with documented clinical history of metal allergy and hypersensitivity reactions		8	7	87.5%	Patients underwent TKA with hypoallergenic implant and had no complications at 12-month post-op review.
	Postoperatively (after at least 6 months)	Patients with painful TKA with referred metal allergy		11	6	54.5%	7 out of 11 patients underwent revision arthroplasty with Nickel-free implant and were free of symptoms and complications at 12-month post-op review.
	Postoperatively (after at least 6 months)	Patients with painful TKA without referred metal allergy or signs of sensitisation		11	2	18.0%	Treated as non-hypersensitive: 3 patients underwent second procedure with Nickel-free implant and pain disappeared. The other patients were treated with analgesics and steroids and had persistent symptoms and variable joint function.
Desai *et al*. [29]	Postoperatively (after at least 3 months)	TKA patients at least 3 months post-op		233	20	8.6%	Patch test positive patients (to all metals) (*n* = 37): 6 patients – pain (*P* = 0.17) 5 patients – loss of function (*P* = 0.03) 5 patients – patient dissatisfaction (*P* = 0.01)
Frigerio *et al*. [30]	Preoperatively	Before TJA (knee or hip)		100	21^a^	21.0%	5 patients with initial negative test (PT or LTT) for MHS became positive postoperatively – 4 were Nickel positive (3 patch test, 1 LTT) 1 patient reported pain without radiographic evidence of implant loosening: No other patients developed cutaneous signs attributable to metal hypersensitivity or implant loosening after TKA or THA.
	Postoperatively (after 1 year)	After TJA (knee or hip)		72	3^b^	4.2%	
Granchi *et al*. [7]	Preoperatively	Control group = no implant, candidates for TKA		20	NR	10.0%	
	Postoperatively (median follow-up 18 months, range 9.6–120)	Stable TKA	All	27	NR	7.4%	
			With clinical symptoms *i.e.* pain	14	NR	7.1%	
			without clinical symptoms *i.e.* no pain	13	NR	7.7%	
	Postoperatively (median follow-up 24 months, range 4.8–132)	Loosened TKA	All	47	NR	23.4%	
			Aseptic loosening	21	NR	23.8%	
			Septic loosening	17	NR	35.3%	
			Mechanical failure	9	NR	0.0%	
Guenther *et al*. [31]	NR	Primary and revision knee and hip arthroplasty patients from historic database		34,914	849	2.4%	
	Postoperatively (mean follow-up 2 years)	Historic database patients with pre-operatively known sensitisation to Chromium, Cobalt, Nickel, or cement component who underwent revision knee (*n* = 14) and hip (*n* = 3) arthroplasty due to a potential allergic reaction ^c^		17	13^a^	76.5%	In TKA patients with likely allergic reactions, Hospital for Special Surgery score (HSS) increased following revision with hypoallergenic coated implants.
Innocenti *et al*. [32]	Preoperatively	Patients with referred or suspected metal allergy before TKA with a hypoallergenic implant		24	21	87.5%	All patients underwent TKA with a hypoallergenic implant. Mean follow-up was 79.2 months (range 61–90). No patients reported any hypersensitivity-related reaction, pain or failure of implant. Postop had improved VAS, KSS and functional score.
Kitagawa *et al*. [26]	Preoperatively	Before TKA		48	3	6.3%	
	Postoperatively (after 6 months)	After TKA with CoCr implant		25	0^b^	0.0%	Both groups showed improved knee score and functional score postoperatively. No clinical or radiological complications observed in either group at 5-year follow up.
	Postoperatively (after 6 months)	After TKA with OxZr implant		22	2 (0^b^)	9.1% (0%)	
Kręcisz *et al*. [27]	Preoperatively	Before TJA (knee [*n* = 21] or hip [*n* = 39])		60	12^a^	20.0%	Patients with confirmed metal allergy preoperatively received implants without sensitising metal, and none developed complications or symptoms postoperatively
	Postoperatively (after 24 months)	After TJA (knee or hip)		48	10^a^/^b^	20.8%	5 patients with newly positive reaction to metal were symptomatic: 3 patients – recurrent pain, swelling and erythema 2 patients – symptoms of metal dermatitis
Lützner *et al*. [42]	Preoperatively	Before TKA with coated (hypoallergenic) implant		61	NR	NR	No patients developed skin reactions or complications with their implant.
	Preoperatively	Before TKA with standard implant		59	NR	NR	
	Postoperatively (after 1 year)	After TKA with coated (hypoallergenic) implant		60	1 'doubtful' reaction^b^	1.7%	
	Postoperatively (after 1 year)	After TKA with standard implant		56	1 'doubtful' reaction^b^	1.8%	
Sasseville *et al*. [33]	Postoperatively (mean follow-up 29.1 months, SD 20.1)	Postop TKA patients with complications		39	4	10.3%	1 PT positive patient underwent revision with 'ceramic' implant and had persistent asymptomatic erythema over knee. 1 PT positive patient underwent revision with titanium implant and had complete remission. 1 PT and LTT positive patient underwent revision with titanium implant and had significant improvement in symptoms. 1 PT and LTT positive patient underwent revision with titanium implant and had no improvement.
Tam *et al*. [34]	Preoperatively	Patients referred for evaluation of MHS before implantation of orthopedic (*n* = 21), cardiovascular (*n* = 7), dental (*n* = 8) and other (*n* = 4) devices (12 TKA patients)		40	17^a^	42.5%	Patients with relevant metal hypersensitivity who underwent revision surgery had complete resolution or improvement of their symptoms, whereas those with metal hypersensitivity who did not undergo revision surgery had persistent symptoms.
	Postoperatively (time frame NR)	Patients referred for evaluation of MHS after implantation of orthopaedic (*n* = 49), cardiovascular (*n* = 4), dental (*n* = 28) and other (*n* = 6) devices (27 TKA patients)		87	14 (6 out of 49 orthopedic implants^a^)	16.10% (12.2% of orthopedic implants)	
Thomas *et al*. [35]	Postoperatively (time frame NR)	TKA patients with yet unexplained complications (loosening, recurrent effusions, and pain)		25	10	40.0%	8 out of 9 patients who underwent revision with hypoallergenic implant reported symptom relief.
	-	"OA-control group" = OA patients awaiting TKA		12	NR	NR	
	-	"PT-control group" = patients without implant but having undergone patch testing for suspected skin allergy		8	NR	NR	
Thomas *et al*. [36]	NR	Patients within study population who had been patch tested in the past for several reasons		48	13^a^	27.1%	
	Postoperatively (time frame NR)	TKA (*n* = 189) and THA (*n* = 61) patients suspected of having allergic reactions with complaints of pain (90.5%), reduced ROM (74%), swelling (67.5%), effusions (29%), loosening (16.5%) and eczema (5.5%)		250	32^a^/^b^	12.8%	
Thomas *et al*. [37]	-	Patients with eczema without metal implant, no CMI		30	0	0.0%	
	-	Patients with eczema without metal implant, with CMI		38	13	34.2%	
	Postoperatively (time frame NR)	TKA (*n* = 47) and THA (*n* = 53) patients without symptoms/complications		100	9^a^	9.0%	
		TKA (*n* = 187) and THA (*n* = 13) patients with symptoms/complications (Pain, effusion, eczema, loosening, reduced ROM)		200	35^a^	17.5%	
Treudler and Simon [38]	Postoperatively (average follow-up NR, range 6–36 months)	TKA patients with suspicion of contact allergy to implant material		13	1	7.7%	The one patient with Nickel hypersensitivity reported pain and palmar eczema.
Verma *et al*. [39]	Postoperatively (time frame NR)	TKA patients with eczema surrounding the knee		15	4	26.7%	All patients were treated with topical corticosteroid resulting in clearing of eczema within 2 weeks. There was no recurrence of eczema or implant complications.
Webley *et al*. [40]	Postoperatively (mean follow-up 2.7 years, range 1–5 years)	Control group = patients with rheumatoid arthritis or osteoarthritis without prostheses		33	NR	NR	
		Patients with hinge arthroplasty of the knee investigated for possible metal sensitivity		50	5	10.0%	33/50 patients—No complications (10 patients had positive PT) 7/50 patients—Loosening (1 patient had positive PT) 10/50 patients—Discharge (5 patients had positive PT)
Zeng *et al*. [41]	Preoperatively	Before TKA		29	NR	7.2%	
		Before THA		67	NR	15.5%	

*Notes. CMI* Cutaneous metal intolerance, *CoCr* Cobalt Chromium, *KSS* Knee Society Score, *LTT* Lymphocyte transformation testing, *MHS* Metal hypersensitivity, *NR* Not recorded, *OA* Osteoarthritis, *OxZr* Oxidised Zirconium, *PT* Patch testing, *ROM* Range of motion, *TJA* Total joint arthroplasty, *TKA* Total knee arthroplasty, *THA* Total hip arthroplasty, *VAS* Visual Analogue Scale

^a^ No information about the break down number per type of prosthesis

^b^ Change in Nickel prevalence when compared to baseline

^c^ Recruited patients with established Nickel hypersensitivity as per their inclusion criteria

Four studies [7, 24, 28, 34] compared the prevalence in different patient groups pre- and postoperatively, and in three of these [24, 28, 34], a lower prevalence was noted in the postoperative cohort. Compared to a control group comprising patients without implants, one study [7] reported a lower prevalence of Nickel hypersensitivity in patients with stable TKA, but a higher prevalence in patients with loosened TKA. Ten studies [25, 29, 31, 33, 35–40] performed patch testing only postoperatively and the prevalence ranged from 7.7% [38] to 83.3% [25].

### Study conclusions and recommendations

Three main themes were commented on: the sensitizing potential of TKA, the relationship between metal hypersensitivity and adverse clinical outcomes, and the utility of patch testing, with the main conclusions summarized in Table 6.

**Table 6:** Main conclusions and recommendations of the included studies

**Sensitizing potential of TKA**		**LE**
TKA may induce metal hypersensitivity.	Kręcisz *et al*. [27]	III
	Desai *et al*. [29]	IV
	Frigerio *et al*. [30]	IV
	Granchi *et al*. [7]	IV
Unable to prove an association between TKA and metal hypersensitivity	Verma *et al*. [39]	IV
Unable to conclude as patients had received hypoallergenic implants	Kitagawa *et al*. [26]	III
**Relationship between metal hypersensitivity and adverse clinical outcomes**		
Metal hypersensitivity may be a cause of complications	Atanaskova Mesinkovska *et al*. [24]	III
	Kręcisz *et al*. [27]	III
	Frigerio *et al*. [30]	IV
	Sasseville *et al*. [33]^a^	IV
	Tam *et al*. [34]	IV
	Thomas *et al*. [37]	IV
	Zeng *et al*. [41]	IV
No relationship between metal hypersensitivity and complications	Carlsson and Möller [25]	III
	Carossino *et al*. [28]	IV
	Granchi *et al*. [7]	IV
	Treudler and Simon [38]	IV
	Verma *et al*. [39]	IV
	Webley *et al*. [40]	IV
**Utility of patch testing**		
Recommend routine pre-operative testing	Kręcisz *et al*. [27]^b^	III
	Desai *et al*. [29]	IV
	Frigerio *et al*. [30]	IV
Only perform preoperatively in patients with a history of metal hypersensitivity	Atanaskova Mesinkovska *et al*. [24]	III
	Kitagawa *et al*. [26]	III
	Carossino *et al*. [28]	IV
	Guenther *et al*. [31]	IV
	Innocenti *et al*. [32]	IV
	Sasseville *et al*. [33]	IV
	Tam *et al*. [34]	IV
Could be a valuable diagnostic tool postoperatively	Atanaskova Mesinkovska *et al*. [24]	III
	Carossino *et al*. [28]	IV
	Desai *et al*. [29]	IV
	Granchi *et al*. [7]	IV
	Thomas *et al*. [35]	IV
	Thomas *et al*. [36]	IV
	Thomas *et al*. [37]	IV
	Zeng *et al*. [41]	IV
	Lützner *et al*. [42]	II
Did not comment on utility of pre- or postoperative patch testing	Carlsson and Möller [25]	III
	Treudler and Simon [38]	IV
	Verma *et al*. [39]	IV
	Webley *et al*. [40]	IV

*Notes. LE* Level of evidence, *TKA* Total knee arthroplasty

^a^ Concluded that whilst possible, metal hypersensitivity was unlikely to be a major contributor to implant failure

^b^ Concluded that patch testing should be mandatory

## Discussion

Nickel hypersensitivity and the implications on TKA is a controversial topic. This systematic review analyses the literature specifically focusing on Nickel hypersensitivity in patients undergoing TKA in order to assess the sensitising potential of TKA, the relationship with clinical outcomes, and the utility of skin patch testing.

### Sensitizing potential of TKA

There was limited evidence to support the concept that implants used in TKA can elicit Nickel hypersensitivity in patients with no prior history of metal hypersensitivity. Only one study [30] which analyzed the prevalence of Nickel hypersensitivity in the same patient group before and after surgery noted that patients developed a newly positive reaction to Nickel after surgery, and this occurred in only three out of the 72 patients available for follow-up (4.2%). The other studies [26, 27, 42] which followed patients up after surgery did not find a significant increase in prevalence of Nickel hypersensitivity following operation, and three studies in the review [24, 28, 34] noted that Nickel hypersensitivity was in fact lower in postoperative patients with implants compared to preoperative patients without implants. Based on the evidence available, TKA implants do not appear to contribute to the development of Nickel hypersensitivity in patients with no prior history of metal allergy.

### Nickel hypersensitivity and clinical outcomes

The literature evaluating the relationship between Nickel hypersensitivity and clinical outcomes was conflicting. Some studies in the review noted that patients with a positive patch test result to a metallic component of their implant developed eczema [39], joint loosening [24], recurrent pain [24, 27], and swelling [27]. They were also more likely to be dissatisfied [29], and a higher prevalence of Nickel hypersensitivity was reported in TKA patients with complications compared to those without [7, 37]. It is conceivable that these symptoms could have been attributable to hypersensitivity, since patients who subsequently had their prosthesis removed, or revised with hypoallergenic implants, experienced resolution of symptoms, whereas those who did not remained symptomatic [24, 28, 34, 35].

Given that up to 20% of patients are not satisfied with the outcome of TKA due to multifactorial reasons [43], it is difficult to ascribe these symptoms to Nickel hypersensitivity alone. Carlsson and Möller [25] followed patients with established preoperative metal allergy up to 16 years after implanting prostheses containing metal to which they were allergic and reported no dermatological or orthopedic complications attributable to contact allergy. Their findings are consistent with other studies which found no significant association between hypersensitivity and pain [29] or radiographic loosening [7, 40]. Furthermore, although Verma et al [39] noted that some patients with a positive patch test developed eczema lateral to the surgical incision, they were unable to correlate their findings, and there is evidence that cutaneous eruptions at this site can develop as a result of resection of the infrapatellar branch of the saphenous nerve when utilizing a medial parapatellar approach [44–46].

### Patch testing

The literature did not support the routine use of preoperative patch testing in all patients undergoing TKA. The majority of studies which commented on the utility of preoperative testing [24, 26, 28, 31–34] suggested that surgeons should consider the overall clinical context, performing patch testing only in patients with a history of metal hypersensitivity, with Granchi et al. [7] reporting that TKA failure was four times more likely in this cohort of patients.

**Fig. 2:**
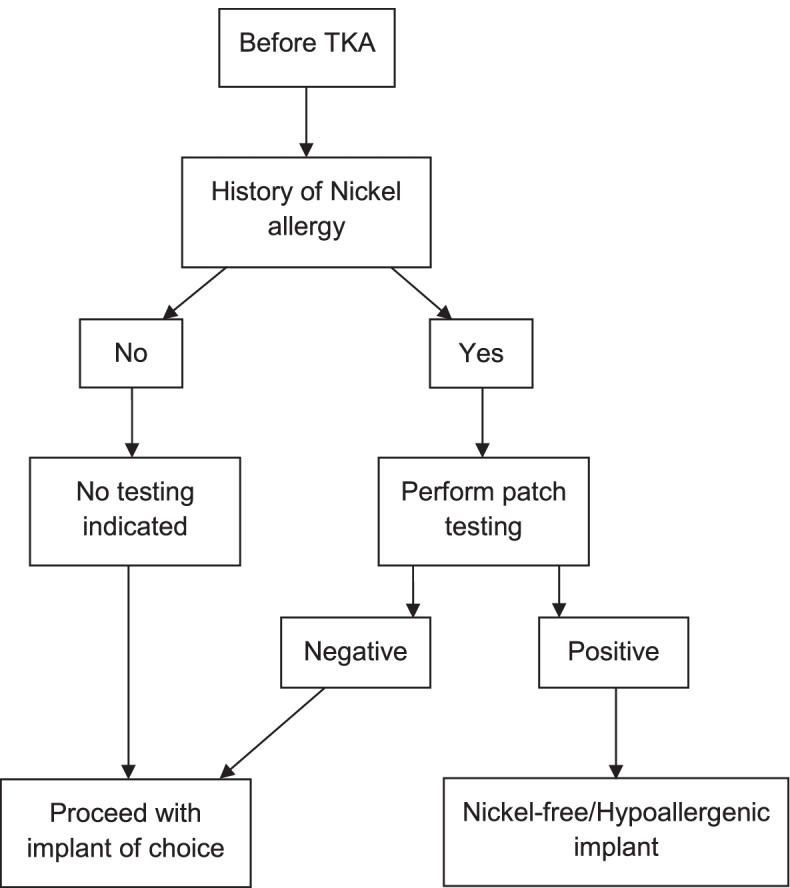
Diagnostic algorithm for Nickel hypersensitivity in patients before total knee arthroplasty (TKA) (adapted from Mitchelson *et al*. [8])

The use of a diagnostic algorithm for metal hypersensitivity in patients undergoing TKA has been proposed in previous articles [6, 9, 14, 15] (Fig. 2). Patients with a positive history of metal hypersensitivity, confirmed with a positive patch test, should be assumed to be hypersensitive to metal and the use of hypoallergenic implants should be considered.

Hypoallergenic TKA implants include coated implants (with Titanium Nitride or Zirconia Nitride), ceramic implants (oxidized Zirconium), pure Titanium implants, and all-polyethylene tibial components [19, 47]. Satisfactory short-to-medium-term outcomes have been demonstrated with these implants. However concerns exist over their longevity and clinical performance [19], so appropriate informed consent should be obtained and shared decision-making should be undertaken.

**Fig. 3:**
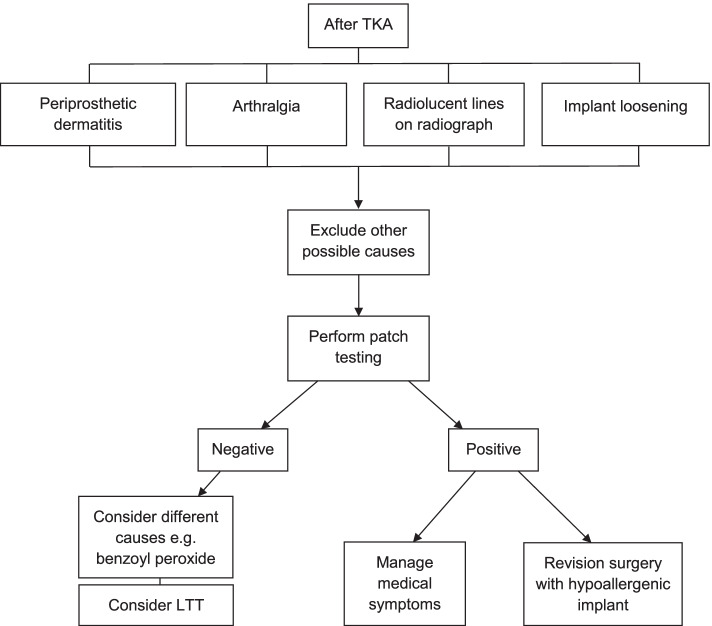
Treatment algorithm for Nickel hypersensitivity in patients after total knee arthroplasty (TKA) (adapted from Mitchelson et al. [8])

The evidence suggests that patch testing could be a valuable diagnostic tool postoperatively to screen for metal hypersensitivity in symptomatic patients following TKA. In patients presenting with recent onset of periprosthetic dermatitis, arthralgia, evidence of loosening, or radiolucent lines on radiographs, patch testing seems a reasonable option once other failure mechanisms such as infection, instability and malalignment have been excluded and inflammatory markers (CRP and ESR) and joint aspiration have yielded negative results [6, 14]. A treatment algorithm could be employed to assist with the management of such patients (Fig. 3). Patients with a positive patch test may have their symptoms treated medically (*e*.*g*. with topical steroids or NSAIDs [6]) or consider undergoing revision with a hypoallergenic implant. This should again involve discussion, shared decision-making and appropriate consenting.

Lymphocyte transformation testing (LTT) was performed in addition to patch testing in a number of studies included in the review [26, 28, 30–33, 35, 37, 41]. LTT detects metal hypersensitivity by measuring the ratio of lymphocyte proliferation in peripheral blood (that has been incubated for seven days) with an antigen present over lymphocyte proliferation with the antigen absent, which is referred to as the stimulation index [10, 48]. It has been suggested that LTT might be more suitable than patch testing as it is more sensitive, less subjective, and patch testing itself can induce metal hypersensitivity in previously non-sensitive patients [26, 28]. However, there are limitations to its large-scale application including cost [29, 42] and the need for specialized laboratories [24]. The role of LTT remains unclear but appears to be gaining support for its use in conjunction with PT when results are negative and allergy remains strongly suspected [33]. Taking synovial biopsies for histopathological analysis of adverse local tissue reactions to implant materials may further assist with diagnosis [19].

Patch testing remains the most commonly used investigation for diagnosing metal hypersensitivity [9, 14, 49]. It is simple, inexpensive, widely available, and may allow for screening of several metals [10, 48] but debate remains over the correlation between dermal reactions elicited by skin patch testing and deep-tissue sensitivity surrounding an implant [16]. Since the primary antigen-presenting cells responsible for contact dermatitis and implant-related hypersensitivity differ [10, 16], it is uncertain whether PT can reliably predict outcomes associated with total knee arthroplasty [6].

### Limitations

This systematic review has several limitations. Firstly, all the included studies had low levels of evidence, with only one [42] scoring above III, based on the OCEBM. In addition, the quality of the studies was poor; none of the observational studies achieving an ideal global MINORS score and the only RCT [42] demonstrating a high risk of bias when assessed using the RoB 2 tool. Caution should therefore be exercised when interpreting and comparing the results of these studies.

Several of the articles analyzed groups of patients undergoing not only knee, but also other orthopedic interventions [24, 27, 30, 31, 34, 36, 37, 41], such as hip and shoulder arthroplasty. However, the results of patch testing in those participants were not stratified by operation, but only as a single patient cohort [24, 25, 27, 30, 31, 34, 36, 37, 50]. Given that specific types of implants, such as metal-on-metal hip prostheses, have a greater propensity to release metal ions and potentially induce hypersensitivity [5], or loosening as the result of a different mechanism from allergy [16], it is difficult to interpret the relationship between Nickel hypersensitivity and total knee arthroplasty in this context.

The utility of late patch test reading at day six after application has been documented [36] and it is possible that many of the studies which interpreted patch tests at day two or three might have missed late positive reactions or been interpreted as false-negative readings. The time until patch testing was performed postoperatively was also highly variable across the studies, and it is thought that shorter periods (*e*.*g*. six months) may be insufficient to detect new hypersensitivity reactions to implant components [51].

## Conclusions

The current literature does not support the concept that patients undergoing TKA with no prior history of Nickel hypersensitivity are at an increased risk of developing hypersensitivity, and there is conflicting evidence that patients with established Nickel hypersensitivity are more likely to experience dermatological or orthopedic complications such as persistent pain, implant loosening or failure. Despite its limitations, cutaneous patch testing remains the most commonly used method for diagnosing Nickel hypersensitivity. The literature does not support routine patch testing of patients prior to TKA but does support performing this test in patients with a history of metal hypersensitivity. In those with a positive patch test, the choice of implant to use should be made on a case-by-case basis after discussion with the patient, as in the absence of more robust evidence, the careful selection of which device to implant may minimize the potential risk of complications related to metal hypersensitivity. Patients with a clinical presentation suggestive of Nickel hypersensitivity following TKA may benefit from patch testing only after the more common causes of pain, loosening and failure have been excluded, since revision surgery with hypoallergenic implants may alleviate symptoms. To further establish the relationship and importance of Nickel hypersensitivity in patients undergoing TKA, large-scale, appropriately designed studies will be required.

## Supplementary Information

**Additional file 1. **Search strategy used in HDAS.

**Additional file 2. **Search strategy used in PubMed.

## Data Availability

All available data are provided. Additional data, if needed, may be made available from the corresponding author on reasonable request.

## Abbreviations

TKA: Total knee arthroplasty
THA: Total hip arthroplasty
PT: Patch testing
PRISMA: Preferred Reporting Items for Systematic Review and Meta-Analysis
MeSH: Medical Subject Heading
HDAS: Healthcare Database Advance Search
LE: Levels of Evidence
OCEBM: Oxford Centre for Evidence-Based Medicine
MINORS: Methodological Index for Non-Randomized Studies
RoB: Risk of bias
RCT: Randomized-Controlled trial
CRP: C-Reactive Protein
ESR: Erythrocyte sedimentation rate
LTT: Lymphocyte transformation testing
