# Supplementary material for: The effect of Nickel hypersensitivity on the outcome of total knee arthroplasty and the value of skin patch testing: a systematic review
Source: Arthroplasty. 2022 Sep 2;4:40. doi: 10.1186/s42836-022-00144-5 (PMC9438335; doi:10.1186/s42836-022-00144-5)
Supplement: Supplementary file 1 — Additional file 1. Search strategy used in HDAS. [file 42836_2022_144_MOESM1_ESM.docx]

**Supplementary Material 1.** Search strategy used in HDAS

|  | Database(s) | Search Term |
| --- | --- | --- |
| 1 | EMBASE | "TOTAL KNEE ARTHROPLASTY"/ |
| 2 | EMBASE | "KNEE REPLACEMENT"/ |
| 3 | EMBASE | "KNEE ARTHROPLASTY"/ |
| 4 | EMBASE | "KNEE SURGERY"/ |
| 5 | EMBASE | "TOTAL KNEE REPLACEMENT ARTHROPLASTY"/ |
| 6 | EMBASE | "TOTAL KNEE REPLACEMENT"/ |
| 7 | EMBASE | "KNEE PROSTHESIS"/ |
| 8 | EMBASE | exp "TOTAL KNEE PROSTHESIS"/ |
| 9 | EMBASE | (1 OR 2 OR 3 OR 4 OR 5 OR 6 OR 7 OR 8) |
| 10 | EMBASE | ("knee surger*" OR TKA OR TKR OR "total knee arthroplast*" OR "total knee replacement*" OR "total knee prosthes*" OR "knee arthroplast*" OR "knee replacement*" OR "knee prosthes*").ti,ab |
| 11 | EMBASE | (9 OR 10) |
| 12 | EMBASE | "NICKEL HYPERSENSITIVITY"/ |
| 13 | EMBASE | "DELAYED HYPERSENSITIVITY"/ |
| 14 | EMBASE | ("nickel allerg*" OR "nickel hypersensitiv*" OR "nickel reaction*" OR "metal allerg*" OR "metal hypersensitiv*" OR "metal reaction*" OR nickel OR metal).ti,ab |
| 15 | EMBASE | (12 OR 13 OR 14) |
| 16 | EMBASE | (9 AND 11 AND 15) |
| 17 | Medline | "ARTHROPLASTY, REPLACEMENT, KNEE"/ |
| 18 | Medline | "KNEE PROSTHESIS"/ |
| 19 | Medline | ("knee surger*" OR TKA OR TKR OR "total knee arthroplast*" OR "total knee replacement*" OR "total knee prosthes*" OR "knee arthroplast*" OR "knee replacement*" OR "knee prosthes*").ti,ab |
| 20 | Medline | (17 OR 18 OR 19) |
| 21 | Medline | HYPERSENSITIVITY/ |
| 22 | Medline | ("nickel allerg*" OR "nickel hypersensitiv*" OR "nickel reaction*" OR "metal allerg*" OR "metal hypersensitiv*" OR "metal reaction*" OR nickel OR metal).ti,ab |
| 23 | Medline | (21 OR 22) |
| 24 | Medline | (20 AND 23) |
