# Supplementary material for: The effect of Nickel hypersensitivity on the outcome of total knee arthroplasty and the value of skin patch testing: a systematic review
Source: Arthroplasty. 2022 Sep 2;4:40. doi: 10.1186/s42836-022-00144-5 (PMC9438335; doi:10.1186/s42836-022-00144-5)
Supplement: Supplementary file 2 — Additional file 2. Search strategy used in PubMed. [file 42836_2022_144_MOESM2_ESM.docx]

Supplementary Material 2. Search strategy used in PubMed

|  | Search Details |
| --- | --- |
| 1 | "arthroplasty, replacement, knee"[MeSH Terms] OR "Knee Prosthesis"[MeSH Terms] |
| 2 | "knee surger*"[Title/Abstract] OR "TKA"[All Fields] OR "TKR"[All Fields] OR "total knee arthroplast*"[Title/Abstract] OR "total knee replacement*"[Title/Abstract] OR "total knee prosthes*"[Title/Abstract] OR "knee arthroplast*"[Title/Abstract] OR "knee replacement*"[Title/Abstract] OR "knee prosthes*"[Title/Abstract] |
| 3 | #1 OR #2 |
| 4 | "Nickel"[MeSH Terms] AND "hypersensitivity, delayed"[MeSH Terms] |
| 5 | "nickel allerg*"[Title/Abstract] OR "nickel hypersensitiv*"[Title/Abstract] OR "nickel reaction*"[Title/Abstract] OR "metal allerg*"[Title/Abstract] OR "metal hypersensitiv*"[Title/Abstract] OR "metal reaction*"[Title/Abstract] OR "nickel"[Title/Abstract] OR "metal"[Title/Abstract] |
| 6 | #4 OR #5 |
| 7 | #3 AND #6 |
